# Examining the relationship between demographic variables and perceived health literacy challenges in Tasmania, Australia

**DOI:** 10.1002/hpja.905

**Published:** 2024-07-29

**Authors:** Madeline Spencer, Nenagh Kemp, Vaughan Cruickshank, Rosie Nash

**Affiliations:** ^1^ School of Medicine, College of Health and Medicine University of Tasmania Hobart Tasmania Australia; ^2^ School of Psychological Sciences, College of Health and Medicine University of Tasmania Sandy Bay Tasmania Australia; ^3^ College of Arts, Law and Education University of Tasmania Launceston Tasmania Australia

**Keywords:** health inequality, health literacy, health promotion, social determinants of health

## Abstract

**Issue Addressed:**

Whilst there is a relationship between health literacy and health outcomes, the importance of social and personal demographic characteristics remains understudied., Identifying the factors responsible for creating health literacy challenges would help ensure that responses are tailored to individual or community needs.

**Methods:**

This survey included questions from five domains from the Health Literacy Questionnaire. Descriptive analyses and hierarchical regression were used to explore the relationship between the demographic characteristics and health literacy. Qualitative questions were subjected to thematic analysis, to identify the current barriers and proposed solutions.

**Results:**

A total of 255 participants completed the survey. Demographic characteristics that significantly predicted lower Health Literacy Questionnaire scores were having one or more *chronic health conditions and living in an area of more socioeconomic disadvantage.* Participants found ‘navigating the health care system’ the most difficult of the five elements measured. A total of 276 individual barriers to health literacy were identified and 162 solutions proposed.

**Conclusions:**

This study provides an overview of our sample's health literacy and how their social demographic characteristics may predict their health literacy challenges. Findings from this study can be used to develop targeted interventions to respond to specific health literacy challenges identified within this population.

**So What?:**

Distinct from other research, whereby solutions are proposed by researchers, the participants in this study were encouraged to share their health literacy challenges and outline potential solutions to address these in their local context.

## 1 INTRODUCTION

A crucial part of creating a healthier society is reducing the burden of illness and preventing disease. Health literacy (HL) is essential to self‐efficacy, disease prevention, and attempts to redress inequities in health.^1^ Determining how HL differs within a population and identifying the perceived challenges to achieving a high level of HL is crucial to creating a healthy society. The individual elements of HL, the social determinants of health, and the impact on health outcomes have been explored over the last decade.^2^, ^3^ However, evidence on the interconnected nature of these three factors is lacking. The present paper explores this gap through mixed methods research and attempts to determine how HL, the social determinants of health, and health outcomes interact within a population.

Health literacy is an emerging concept, and its definition is continuously expanding and changing. One definition of health literacy, and the one used for this paper, is ‘health literacy represents the personal knowledge and competencies that accumulate through daily activities and social interactions and across generations. Personal knowledge and competencies are mediated by the organizational structures and availability of resources that enable people to access, understand, appraise, and use information and services in ways that promote and maintain good health and well‐being for themselves and those around them’.^4^

It is clear from this definition that HL brings together a number of concepts that relate to both the individual‐ and population‐level assets required to make effective decisions about health for themselves, their families, and their overall communities.^5^, ^6^, ^7^ HL is an important element of a person's overall health. International organisations such as the World Health Organisation (WHO) (2015) are currently focused on ways to improve, adapt, and bolster the HL assets of individuals and organisations.

In developed nations, including Australia (where this research was conducted) most people will acquire a communicable disease (CD) (e.g., COVID‐19) during their lifetime.^8^ However, non‐communicable Diseases (NCDs) are the leading cause of death and disability, accounting for 71% of all deaths globally.^8^ In Australia, NCDs are responsible for 89% of all deaths and place a significant burden on the health system.^9^ NCDs are also referred to as chronic diseases and tend to be of long duration. They are the result of a combination of genetic, physiological, environmental, and behavioural risk factors^10^ and include conditions such as cardiovascular diseases, cancers, chronic respiratory diseases, and diabetes. Their risk factors, such as unhealthy diets, physical inactivity, exposure to tobacco smoke or the harmful use of alcohol, are modifiable.^10^ The escalating burden of all diseases continues to contribute to the ‘social gradient’ in health outcomes^2^ and is amplifying health inequity and poverty worldwide.^11^

Health promotion and health education aim to assist individuals to improve and take control of their own health. Ensuring both translate into the development of health literacy assets is imperative.^3^, ^12^ Health is seen as a resource for everyday life, and therefore an individual or group must be able to identify and realise aspirations, satisfy needs, and change to cope with their environment. By creating systems and environments that can support people to make healthy choices, we may prevent NCDs from developing, mitigate the spread of CDs, and reduce the impact of both on population health outcomes.^13^ In Australia, the National Preventative Health Strategy (2021) has been developed to improve the health and wellbeing at all stages of life. This Strategy uses a systems‐based approach to prevention that addresses the wider determinants of health, reduces health inequities, and decreases the overall burden of disease.^14^ However, in isolation, health promotion and disease prevention are not enough to empower individuals and communities to take an active role in improving their health. Health promotion efforts must be tailored and responsive to the existing population's health literacy strengths and challenges to help individuals develop the assets they need to make autonomous decisions regarding their health and wellbeing.^15^ In Australia, the Commission of Safety and Quality of Health Care (2014) has created a national action plan to address HL via a coordinated approach to increase the safety, quality, and sustainability of the health care system.

Improving HL is one important strategy required to reduce the unsustainable stress on health care systems. HL has been defined as a social determinant of health (SDH) in its own right.^16^ In their exploration of HL as a determinant, mediator and/or moderator of health, Pelikan et al. (2018) concluded that comprehensive HL is a critical, direct determinant of health. They highlighted that HL has considerable potential for health promotion and may improve population health to tackle health inequity. The SDHs are described as the non‐medical factors that can influence health outcomes. They are the conditions in which individuals are born, grow, live, work and age, and they can influence the prevalence, distribution, and risk of developing disease.^2^ Disadvantaged social and socio‐economic conditions can contribute to low HL levels and poorer health outcomes.^17^ Addressing the common risk factors and SDH is an important step in reducing the growing burden of disease in Australia and globally.^18^ The SDHs link closely to an individual's demographic characteristics and can thus be predictors of HL challenges. Previous research has identified a range of factors that can influence someone's overall HL, including gender, household make‐up, education level, employment status, identification as an Aboriginal or Torres Strait Islander, and speaking languages other than English at home.^19^, ^20^, ^21^ For research purposes, it is often simpler to collect a person's demographic profile rather than to try to determine their SDHs.

In Australia, the National Health Survey (NHS) collects data on the health of Australians, including health conditions, health risk factors, and all domains of the Health Literacy Questionnaire (HLQ).^22^ In 2017–2018 the NHS reported that people living in the island state of Tasmania have some of the lowest HL scores nationally on a selection of HLQ domains. The NHS (2017–2018) only looked at the HLQ scores and did not expand to include specific demographic data.^22^ This is important for those involved in HL promotion, given that having low HL can result in poor health outcomes.^23^ However, low HL may not always be a consequence of the individual's assets and the HLQ is designed to also acknowledge the contributions made by the health services and system.^6^, ^7^ Extending on this thinking, Ophelia which stands for ‘OPtimising HEalth LIteracy and Access’ provides an innovative process that focuses on a whole‐of‐system approach to developing grounded health literacy interventions that respond to local community strengths and challenges.^24^ Therefore, the HLQ serves as an assessment tool within the Ophelia framework, which supports the collection of individual's HL across multiple domains and therefore the health literacy strengths and challenges at a community level. The Ophelia principles aim to enhance HL by focusing on organisation, staff, communication, environment, and access. There are eight Ophelia principles: Outcomes focused, Equity driven, Needs diagnosis, Co‐design, Driven by local wisdom, Sustainable, Responsive and Systematically applied. The Ophelia process involves seven steps: assess the health literacy needs, develop a plan, organise the plan, implement interventions, evaluate outcomes, reflect and sustain, and communicate findings.^24^

As noted above, Tasmania experiences some of the worst health outcomes nationally. When comparing the rates of NCDs in the Tasmanian population to those of the nation, there is a greater prevalence of heart disease, diabetes, airway diseases, and cancers.^22^ Tasmanians also perform poorly against several NCD risk factors, including fruit and vegetable consumption, cigarette smoking rates, and obesity.^22^ Tasmania's growing health concerns have been recognised and government departments are taking steps to address them. The government initiative: *Tasmanian Health Literacy Action Plan*,^25^ has been designed to improve the health of the population. A recent report (2022) which considered the Tasmanian and Australian HLQ data highlights the specific strengths and challenges experienced by people in our communities.^26^ Whilst this report was not available when we commenced our research it outlines a set of important recommendations. Fortunately, in response to their findings, the interventions outlined in the report focus mainly on the ‘navigation’ aspect of HL. However, it may be helpful to extend into the other HL domains where the local population reported experiencing difficulties. As this will require continued effort, more research on local strengths, needs, and preferences will be essential to build upon the current recommendations into the future.

In recognition of the HL disparities and significant burden of NCDs in the region, this study aimed to use the HLQ to assess how HL scores differ with social demographic characteristics. As described, we anticipated a social gradient of health literacy and health outcomes to exist in Tasmania. However, there is very little existing evidence about the factors (e.g., education, income, social supports) which determine, mediate, and moderate an individual's HL in Tasmania. This exploratory study represents the second study of a six‐stage broader research project and was designed to help identify which sociodemographic characteristics (including income, education status, and physical health) are associated with people's greatest self‐reported HL challenges. This research represents the first phase of an Ophelia study that is specifically focused on informing a future Health Literacy Mediator (HLM) role for communities. This HLM role may be able to address health inequity experienced by some members of our community.

## 2 METHOD

### 2.1 Sampling and participants

This study was conducted using a cross‐sectional exploratory design and invited people living in the state of Tasmania, Australia to participate. Following ethics approval from the University of Tasmania Human Research Ethics Committee (H0026170), a convenience sampling approach was used. Participants were recruited between October and December 2021 through email and social media advertising, as well as via discussions with HL networks/organisations and community services (including services supporting migrants and adult literacy/numeracy development). Participants were eligible to participate if they were 18 years of age and over and residing in Tasmania.

### 2.2 Data collection

The Health Literacy Questionnaire (HLQ) was used to collect HL data. The HLQ is a multi‐dimensional assessment tool used to identify HL strengths and challenges.^27^ It was developed following a validity‐driven approach utilising data from the general population, patients, health care providers, and policy makers.^22^ The HLQ contains 44 questions relating to 9 domains^6^:

*Domain 1*—Feeling understood and supported by health care providers
*Domain 2*—Having sufficient information to manage my health
*Domain 3*—Actively managing my health
*Domain 4*—Social support for health
*Domain 5*—Appraisal of health information
*Domain 6*—Ability to actively engage with health care providers
*Domain 7*—Navigating the health care system
*Domain 8*—Ability to find good health information
*Domain 9*—Understanding health information well enough to know what to do

These domains are applicable to all individuals throughout their life course and take account of both the challenges and the needs of society. The first five domains (1 through to 5) have responses ranging from 1 = *strongly disagree* to 4 = *strongly agree*, while the remaining domains (6 through to 9) have responses from 1 = *cannot do or always difficult* to 5 = *always easy*. It should be noted that the HLQ is not designed to allow the calculation of an overall score.^6^ Instead, the scores for each individual domain were examined to identify potential HL challenges and strengths. Each domain is considered to be an independent entity, and therefore researchers can choose to focus on one or more of the nine domains to answer specific research questions and evaluate specific outcomes.^27^

The research team discussed all nine domains and carefully selected five domains for this research. The specific domains chosen were intended to enhance our insights into the interactive and social ecological nature of HL, which is important for addressing the research questions. The domains selected for inclusion: 2, 3, 4, 6 and 7,^i^ were those that assessed individuals managing their own health, and relationships that individuals develop when interacting with the health care system or their social supports to make health‐related decisions each day. This approach made it possible to explore that interaction point, so that we could go further than just a focus on an individual's HL. This specific focus ensured greater alignment with the Ophelia principles of being equity‐driven, listening to local wisdom and using a needs assessment to inform responsive health and community services.^7^

The two qualitative questions in this study asked participants:

1. What challenges/barriers have you experienced in the last 12 months when utilising health information, health care, and community health services?
2. Can you think of anything that would help you overcome each of these barriers?

Participants provided their own freeform answers with no limits on the number of discussion points. These barriers and proposed solutions will inform the roles and responsibilities of a HLM. This is out of scope of the current paper, but will be expanded upon in future papers.

An online approach was selected due to the ongoing constraints of the COVID‐19 pandemic and subsequent lockdowns at the time of data collection. Participants completed an anonymous survey, which collected quantitative and qualitative data via REDCap (Research Electronic Data Capture), a secure web application.^28^ Before participants could progress to the survey questions, a written information leaflet that explained the study, its purpose and the potential benefits was made available and participants were required to provide electronic informed consent. Participants provided demographic information about their age, gender, country of birth, Aboriginal or Torres Strait Islander (ATSI) status, education, employment, and self‐reported number of chronic health condition/s or disability status.

### 2.3 Data analysis

All data gathered from the surveys were analysed using jamovi 1.6.23. Descriptive statistics were calculated, and a series of hierarchical regression analyses were conducted to determine which of the demographic variables could account for the variance in the five individual HLQ domain scores assessed. For this analyses, the general assumptions were tested first. Quantile‐quantile plots revealed that the distribution of errors was acceptable, and the Variance Inflation Factor (VIF) values of less than 10 and tolerance values greater than .02 indicated no significant issues with multivariate collinearity.^29^ In each analysis, the variables were broken down into three distinct blocks and a three‐stage hierarchical regression was run.

Thematic analysis, using Braun and Clarke's (2006) six‐phase methodology, was used to analyse participants' qualitative comments that identified potential barriers and solutions to their utilisation of health information, health care, and community health services. Thematic analysis was chosen to identify recurring themes within the responses that might reveal meaningful patterns. Some participants provided a single barrier or solution, whilst others listed as many as three. To maintain rigour, after Author 1 completed the analysis, Author 2 independently recoded 5% of the themes, and a comparative discussion occurred, prior to the final themes being decided.

## 3 RESULTS

A total of 255 participants returned complete responses to the online survey and were included in the analyses. (A further 15 participants were excluded for completing none (*n* = 5) or less than half (*n* = 5) of the questions.) The demographic characteristics for the study sample can be found in Table 1. Approximately 80% of participants identified as female, the majority were highly educated (71%), holding a bachelor's degree or higher, most were from solely English‐speaking households (96%), and Australian citizens (88%). However, some variation in age and education was observed, and over half of participants (54%) reported having at least one chronic health condition.

**TABLE 1:** Participant demographic characteristics.

Demographic variable	Total (*N* = 255)	
	*n*	Percentage
Gender (*n* = 255)		
Male	46	18
Female	204	80
Non‐binary	3	1.2
Prefer not to answer	2	.8
Age (*n* = 247)		
18–35 (young adult)	65	26.3
36–55 (middle aged)	123	49.8
56+ (older adult)	59	23.9
Nationality (*n* = 246)		
Australian	215	87.4
International	31	12.6
Index of Relative Socio‐Economic Disadvantage (IRSD) (*n* = 244)		
1 (most disadvantaged)	33	13.5
2	55	22.5
3	49	20.1
4	50	20.5
5 (least disadvantaged)	57	23.4
Relationship (*n* = 255)		
Single	46	18
In a relationship	50	19.6
Married/De Facto	156	61.2
Other	3	1.2
Household composition (*n* = 255)		
Living alone	34	13.4
Living with others	221	86.6
Language spoken at home (*n* = 254)		
Only English	244	96
Additional languages	10	4
Indigenous/Torres Strait Islander (ATSI) (*n* = 254)		
Yes	14	5.5
No	240	94.5
Education status (*n* = 254)		
High school	26	10.3
TAFE/Trade	46	18.1
University undergraduate	111	43.7
University postgraduate	71	27.9
Employment status (*n* = 255)		
Working full time	110	43.2
Working part time	91	35.7
Home duties	7	2.8
Full time/part time student	10	3.9
Retired	22	8.6
Unable to work	2	.8
Other	13	5
Number of chronic health condition/s or a disability (*n* = 252)		
0	114	45.3
1	81	32.1
2+	57	22.6

Descriptive statistics were calculated for each of the HLQ domains and can be found in Table 2. A higher average score implies less challenges, whereas a lower average score implies more challenges. Whilst these findings cannot be compared directly, these results are tabulated alongside data from the Australian Bureau of Statistics (ABS) data collected in the 2017–2018 NHS.^22^ It was found that Tasmanians had numerically lower scores and so potentially faced more HL challenges across all assessed domains than the national averages in the NHS. It should be noted, however, that without accessing the ABS complete data set, we cannot analyse this finding further to assess significance between this study and the Tasmanian and national ABS data.

**TABLE 2:** Descriptive statistics for each Health Literacy Questionnaire (HLQ) domain.

HLQ domain	Mean (RSE)		
	Study population	Tasmania^a^	Australia^a^
Domains with a possible total score of 1–4 (*n* = 240)			
2) Having sufficient information to manage my health	3.10 (0.6)	3.14 (0.8)	3.17 (0.3)
3) Actively managing my health	3.05 (0.5)	3.06 (0.7)	3.09 (0.3)
4) Social support for health	3.02 (0.6)	3.14 (0.9)	3.19 (0.3)
Domains with a possible total score of 1–5 (*n* = 232)			
6) Ability to actively engage with health care providers	3.74 (0.7)	4.13 (0.9)	4.18 (0.3)
7) Navigating the health care system	3.44 (0.7)	3.97 (0.9)	4.02 (0.3)

Abbreviation: RSE, relative standard error.

^a^ Data sources from: ABS (2019c).

Summaries of the regression statistics for the final regression models that included statistically significant demographics are presented in Table 3. The two demographic characteristics that significantly predicted outcome variables (HLQ scores) were (1) having one or more chronic health conditions and (2) living in an area with a low Index of Relative Socio‐Economic Disadvantage (IRSD). In order to carry out the hierarchical regression, chronic conditions included the categories of 0, 1 or 2 or more chronic conditions, while IRSD included numerical values 1–5 (corresponding with the index of relative socioeconomic disadvantage where a low score indicates relatively greater disadvantage). These predictors were found to be the strongest in the domains of ‘Social support for health’ and ‘Navigating the health care system’, accounting for 15.3% and 13.8% of the variance respectively. Meaning that the more chronic health conditions an individual had were associated with lower HLQ scores and living in a lower IRSD are also associated with lower HLQ scores. No further factors explained a significant amount of variance for our participants.

**TABLE 3:** Significant hierarchical regression results of the final regression model when predicting HLQ scores.

Group	*β*	*t*	*R*	Adjusted *R* ^*2*^	*F*	*p*
Having sufficient information to manage my health (Domain 2)			.23	.04	6.08	.003
Chronic health conditions	.41	3.08*				
IRSD	.09	1.43				
Actively managing my health (Domain 3)			.21	.03	4.81	.009
Chronic health conditions	.07	.56				
IRSD	.20	3.01*				
Social support for health (Domain 4)			.40	.15	20.81	<.001
Chronic health conditions	.64	5.14*				
IRSD	.22	3.55*				
Ability to actively engage with health care providers (Domain 6)			.29	.09	10.13	<.001
Chronic health conditions	.41	3.10*				
IRSD	.19	2.99*				
Navigating the health care system (Domain 7			.38	.14	17.92	<.001
Chronic health conditions	.46	3.60*				
IRSD	.28	4.46*				

*Note*: *β* is standardised coefficient, adjusted *R* ^2^ is coefficient of determination.

* *p* < .05.

Thematic analysis of the participants' qualitative comments revealed the current challenges that individuals face when utilising health information, health care, and community health services. Overall, the 255 participants identified a total of 276 barriers, and came up with a total of 162 solutions. The main themes identified during analysis of the qualitative data can be found in Table 4. Themes that emerged in participants' comments regarding barriers were, from most to least commonly mentioned: (1) Availability and access to health care, (2) Lack of perceived support from the health care system, (3) Difficulty understanding and navigating health, and (4) Expense of health care. The themes which arose from the participants' identified solutions to the above barriers were, again in order from most to least commonly identified: (1) Increased availability and access to health care providers/services, (2) Better collaboration between primary health care and other services, (3) Subsidised health care services and funding and (4) Health promotion.

**TABLE 4:** Explanations and proportions of responses per theme, for the barriers and solutions regarding utilisation of health information, health care, and community health services.

	Theme	Proportion	Explanation and example quote(s)
*Barriers* (Total identified 276)	Availability and access to health care	.62	Participants noted that they often struggled to access appointments to see health care professionals in a timely manner, for example, ‘*It has become very difficult to see my regular GP when I need too [*sic*]. It is also difficult to see other practitioners within the same practice when I need to. The poor availability of health services, especially highly specialised ones mean that current health care is not servicing the community*.’ Most of participants commented that access to specialist care was lacking within their communities, especially when they lived outside of the major metropolitan areas.
	Lack of perceived support from the health care system	.19	Some participants did not feel that they were being listened to and supported whilst on their health care journey, for example, *‘Health professionals do not listen to me’* or *‘Health Professionals are not forthcoming with information and not good communicators’*.
	Difficulty understanding and navigating health	.11	Ongoing challenge of both navigating and understanding the health system itself but also the information that is provided by the system, for example, ‘*I notice this particularly in my work with young people they struggle to understand how to access services—and there is not enough education available to help them learn how to navigate the health care system* and *I feel that I am often considering how hard it is to get health information to allow me to make informed decisions about my health’*.
	Expense of health care	.08	This barrier was leading individuals to avoid the system all together as the costs involved were simply beyond their daily living budgets and current government subsidies did not go far enough. This issue was amplified when specialist care was required. For example, one participant explained that they *‘experienced a cost barrier when seeking treatment for ongoing health issue, procedure was not covered by insurance or private health’*.
*Solutions* (Total identified = 162)	Increase availability and access to health care providers/services	.53	Participants suggested that an increase in the availability of services would help with their current health journey. For example, ‘*Either increasing the number of GPs in Tasmania or alleviating the existing GP workloads by diverting those clients that could be diverted to other health care options if they were available here (many free nurse/allied health/mental health clinics in other parts of the world but are not available here, leaving doctors as only option)’*.
	Better collaboration between primary health care and other services	.20	Improved collaboration was suggested so that those involved with a patient's care were well informed and could work together to provide appropriate care. Suggestions included: ‘*Service providers working in collaboration with each other’* to *‘a health coordinator in the region who can advocate or concierge to help navigate the public health system’*. Another common idea was to have *‘a digital platform for clinic appointments, to show where you are in waiting lists, and where health information is available’*.
	Subsidised health care services and funding	.14	This theme involved the idea of making health services more affordable to the community. Many noted that more funding was required all round, or more specifically, that *‘Providing the right incentives for doctors (and disincentivise quick visits and insensitive deeper conversations) would likely save the health care system money over time as people provided proper care plans from the start and so would no need to represent as often’*.
	Health promotion	.13	Another solution suggests by the participants was to improve people's current relationship with the health care system. Suggestions ranged from: ‘*A specific service that specialises in health literacy of vulnerable communities and provides education to those at risk is highly needed and would be extremely beneficial*’, to ‘*More written and visual information about processes and promotion of available about services, or medical conditions’*.

## 4 DISCUSSION

The aims of this study were to describe current health literacy levels on five of nine domains of the HLQ and to assess how HL scores differ with social demographic characteristics in a Tasmanian sample. The HLQ was used to identify local HL strengths and challenges, while the qualitative questions identified barriers and proposed solutions regarding utilisation of health information, health care, and community health services. Participants with more HL challenges (lower HL scores) were those who had chronic health conditions or who lived in areas of disadvantage. Interventions focusing on the HL of individuals and the HL responsiveness of services may be able to overcome these barriers by incorporating some of these solutions provided by the local community.^7^ Other services or organisations may find the Ophelia process (health literacy assessment process) helpful to ensure their services respond to the HL strengths and challenges of their local community.

Although it was not a specific aim of this study, the HLQ results in this study can be compared to those described in the NHS for both the Tasmanian and Australian populations. As expected, the mean scores in the current Tasmanian data set were numerically lower on all five domains than the national data. This indicates that Tasmanians may have more challenges than their national counterparts when it comes to actively managing their health, having access to sufficient information, and having enough social support for their health. This finding aligns with the 2018 HLQ data presented in the *Optimising Health Care for Tasmanians* report.^26^ The report outlines several recommendations, which include: Understanding and responding to health literacy diversity; Optimising the reach and impact of health messaging; Improving health literacy responsiveness; Co‐designing health literacy actions; Local Ophelia projects; Evaluation and monitoring.^26^ Given the finding that IRSD had an important relationship with the HL strengths and challenges of our participants, our research reinforces the importance of understanding and responding to health literacy diversity, specifically taking into account the IRSD of the individuals and community the health literacy intervention is being designed to support. Further, if co‐design is employed to obtain and include local wisdom in the development of solutions, then it is important to consider how IRSD may impact meaningful contributions. The recommendation from the Optimising Health Care for Tasmania report that health literacy responsiveness be improved is an important one, and our participants reported that they, too, would like to see ‘health care servicing the community’, not the individuals having to work for the care they deserve. Finally, this study provides an example of a local project that follows the Ophelia process to support Tasmanians to understand their health literacy strengths and challenges and generate data‐driven solutions.

Whilst the current study's participants were relatively highly educated compared to the ABS data set outlined in the *Optimising Health Care for Tasmanians* report, our sample is still experiencing certain HL challenges. Those individuals who were less likely to respond and didn't respond to our survey (e.g., minority groups and those of a lower education status) are likely to experience more HL challenges and thus may require significantly more support.^30^, ^31^ This has important implications for policy makers and health professionals providing services to the Tasmanian community. Specifically, this body of research will inform the future development of a HLM codesigned with local community to address the inequities that exist.

The regression models demonstrated that having one or more chronic health conditions accounted for the largest proportion of the variance in terms of HL challenges, as reflected across four out of five of the HLQ domains assessed. There are two ways in which this finding could be interpreted. First, it is possible that greater HL challenges mediate the development of chronic diseases. An alternative explanation is that when people suffer from chronic diseases, they rely on their current HL assets to manage their conditions, and thus challenges are more readily identified. These ideas have been discussed elsewhere in the literature. For example, Friis et al. (2016) found that people with long‐term health conditions reported more difficulties than the general population in understanding health information and actively engaging with health care providers. Other authors have concluded that HL plays a crucial role in chronic disease management and prevention.^32^, ^33^ Another consideration is that people with chronic health conditions must navigate the health care system relatively often, and thus may encounter barriers and difficulties that are not encountered by those without chronic disease. One study concluded that HL is not just an individual skill but is also highly dependent on the accessibility of the health care system, the communication skills of health care professionals, and the level of complexity of the health information.^34^ Therefore, for a HL intervention specific for chronic conditions, it is important that a multidisciplinary team of health professionals work together with community members to provide ongoing support and care. The strength of a team approach and its provision of a range of different skills can meet the varying needs of individuals, particularly in relation to their values and perspectives on their chronic conditions.^24^

The regression models demonstrated that greater challenges across HLQ domains were also predicted by living in an area with greater relative socio‐economic disadvantage. It has been identified that disadvantaged social and socio‐economic conditions contribute to low HL levels and poorer health outcomes.^35^, ^36^ Furthermore, by definition, areas of low IRSD also have poor levels of educational attainment, which is an important determinant of HL.^37^ In 2021, Schillinger took this concept one step further and created a framework that describes two primary pathways that generate consequences for health outcomes, based in part, on HL.^38^ Tasmanian research on this topic has pointed to the HL challenges (mainly around access) that people are facing in low IRSD areas as a contributing factor in preventable hospital admissions for patients suffering from chronic health conditions.^39^

**FIGURE 1:**
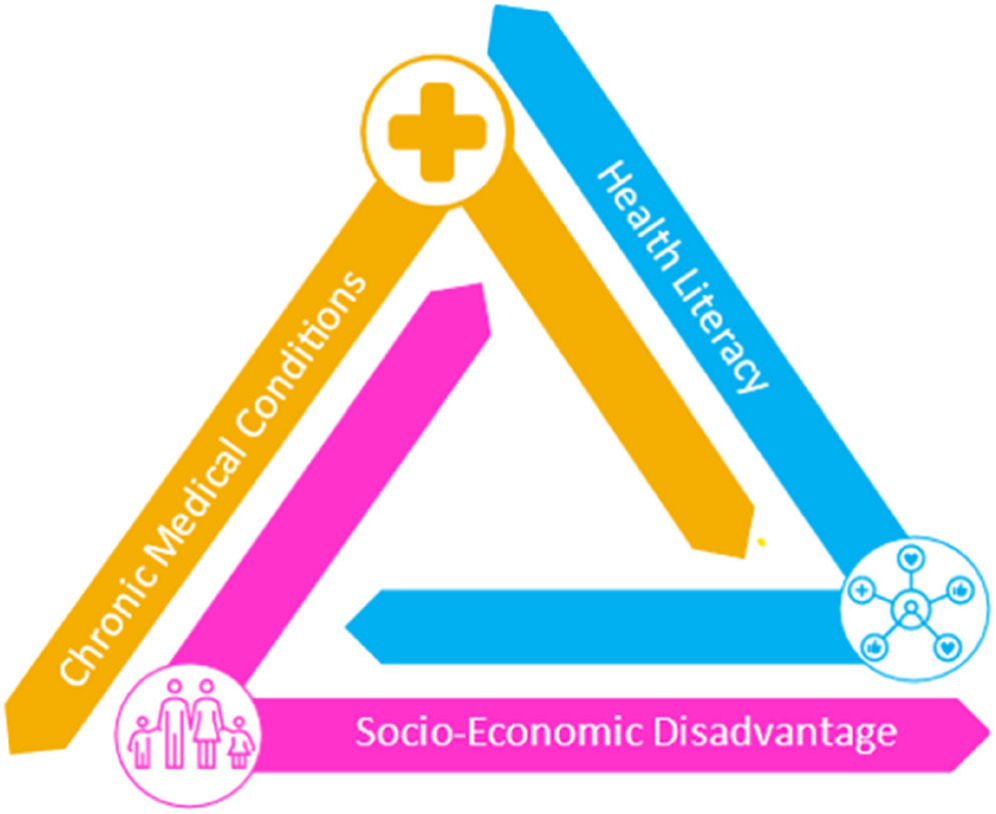
Tri‐directional relationship between socio‐economic disadvantage, chronic health conditions and health literacy.

This research has alluded to a connection between HL, chronic health conditions, and socio‐economic disadvantage. This relationship, which has been shown in Figure 1, is well attested to in the literature and is referred to as the ‘social economic gradient of health’.^40^, ^41^ Here, there is a tri‐directional relationship between these factors, meaning that they are all related to, and influence, each other. For example, Tasmanians living in areas of low IRSD have more challenges associated with obtaining, processing, and understanding basic health information and services needed to make appropriate health decisions. This in turn increases both their risk of developing a chronic disease, and the severity of that disease.^42^, ^43^ This is especially problematic because these people are more likely to experience worse health outcomes, inadequate educational attainment and use health services suboptimally.^2^, ^19^, ^44^ It could be argued that these individuals are most in need of services and support. However, the way the health system is currently designed does not specifically target or make services more accessible to such groups. These challenges are mirrored in other regional areas within Australia.^45^ Further, this set of problems has also been described in the international literature and raised as an issue that deserves attention by many experts in this field.^46^, ^47^

Other demographic characteristics have been noted in the literature as predictors of HL challenges. These include level of education, employment status, identification as an Aboriginal or Torres Strait Islander, and speaking languages other than English at home.^19^, ^20^, ^21^ Due to the number of participants and the relative lack of variety in participants' demographics in the current sample, some of these other important demographics were not demonstrated, probably because there was not enough representation to be able to find significant results. Although these characteristics did not contribute significantly to predicting HLQ scores in our participants, it is still critical that they are not ignored. For example, higher educational attainment is associated with higher HL.^19^, ^48^ It is known that higher educational attainment can then play a significant role in mediating the relationship between HL and health behaviours.^48^

Consistent with the Ophelia principles, when seeking to overcome barriers associated with HL it is essential to consider the lived experience and context of the individual.^7^ We identified a number of barriers from participants' responses about interacting with health system: availability and access to health care, expense of health care, lack of perceived support from the health care system, and difficulty in understanding and navigating health. Current literature has recognised a similar set of barriers.^39^, ^41^ We also identified a set of solutions emerging from participants' responses: the need for increased availability and access to health care providers/services, subsidised health care services and funding, better collaboration between primary health care and other services, and an emphasis on health promotion. Each strategy may be utilised to inform change or reform to the health system. This will require a systems approach.^49^

The multidimensional nature of HL means that it requires a collaborative approach. As such, those in health, education, and community sectors could benefit from working more collaboratively. The results from this study have highlighted the disparities in HL assets amongst people within one community. Consistent with the Ophelia principles that informed this research, these results could be used to guide future investigations into the most effective interventions to assist in enhancing the HL assets of individuals and communities to improve overall health. A prominent finding of the study is that suffering a chronic health condition or living in an area of low IRSD were predictors of experiencing greater HL challenges across all measured HL domains. This result suggests that interventions aimed at addressing HL and health inequities would do well to be situated in disadvantaged communities and be made available and accessible to those who live with chronic health conditions.

Current interventions and strategies at the local, national, and international level have been implemented in various forms. These strategies acknowledge the importance of HL, and its centrality to health inequalities, given that disadvantaged groups are most at risk of poor health behaviours and outcomes.^50^ All of these interventions aim to increase people's control over their own health by assisting them to understand and traverse the health care system to decrease health risk factors and improve health outcomes. However, these initiatives do not comprehensively address all the challenges outlined by our participants. As identified in this paper, strategies, interventions, and policy reform should focus on community members living with socio‐economic disadvantage, chronic health conditions or both (as described by the tri‐directional relationship). New initiatives could also address the barriers regarding utilisation of health information, health care, and community health services, which include enhancing the availability of services, providing assertive outreach style support, and developing community understanding of health care and the health care system. A wide range of stakeholders have an important role to play in strengthening HL, as identified by the International Union for Health Promotion and Education (IUHPE) Position Statement on Health Literacy, which emphasises the necessity of a systems approach to HL, underpinned by global, national, regional and local policies.^16^

Results from this study do need be interpreted with some degree of caution. Participants were recruited via a convenience sampling approach, which led to limitations with regards to the sampling and demographics of participants. We approached local community groups and organisations including the Migrant Resource Centre, 26TEN (an organisation that supports adult numeracy and literacy development), and the Tasmanian Health Literacy Network to encourage broad participation throughout Tasmania. Despite these efforts, the demographic characteristics were still significantly skewed. Specifically, the majority of participants were Australian‐born, English‐speaking, tertiary‐educated, middle‐aged females. This indicates that the survey was unable to capture the responses of many members of important minority groups, such as culturally and linguistically diverse (CALD) communities. Thus, the results are not representative of the experiences of all members of the Tasmanian community. Future research should aim to recruit a larger proportion of these underrepresented groups (e.g., telephone survey, in‐person survey support, translator assisted survey completion).

Future work could also use a longer survey to encompass all domains of the HLQ. These limitations were also exacerbated due to the data collection process being conducted during a time where the impacts of the COVID‐19 pandemic were still being felt across Australia. Consequently, the potential for pandemic‐related impacts on HL assets should not be disregarded. As this research is just one part of a larger project following the Ophelia approach, subsequent papers will use these finding to formulate case‐based discussions to inform a future role of a Health Literacy Mediator for the community.

## 5 CONCLUSION

In the small island state of Tasmania, individuals from different communities are experiencing ongoing challenges when it comes to the utilisation of health information, health care, and community health services. This alone can contribute to poorer health outcomes for both the individual and their community. This paper identified that those living in areas of greater socio‐economic disadvantage face greater HL challenges than their peers across the HLQ domains of 3, 4, 6 and 7. Whilst those living with one or more chronic health conditions face greater HL challenges for the HLQ Domains 2, 4, 6 and 7. Finally, the results help to identify the participant‐identified key barriers and potential solutions to the local population's health challenges. These findings could be used to develop targeted HL interventions designed to respond to the HL strengths and challenges identified within this population.

## FUNDING INFORMATION

This research was made possible by a PhD scholarship from the College of Health and Medicine from the University of Tasmania awarded to M. Spencer. All the other authors have received no financial support for the research, authorship, and/or publication of this article.

## CONFLICT OF INTEREST STATEMENT

Dr R Nash is Editorial Board member of Health Promotion Journal of Australia and co‐author of this article. To minimise bias, they were excluded from all editorial decision‐making related to the acceptance of this article for publication. Other authors have no conflicts of interest declared.

## ETHICS STATEMENT

University of Tasmania Human Research Ethics Committee (H0026170).

## Data Availability

The data that support the findings of this study are available from the corresponding author upon reasonable request.

## Endnote

^i^

*Domain 2*—having sufficient information to manage my health, *Domain 3*—actively managing my health, *Domain 4*—social support for health, *Domain 6*—ability to actively engage with health care providers, and *Domain 7*—navigating the health care system.
